# 64. Absolute Monocyte Count (AMC) as Early and Safe Marker for Discharge in Low-risk Pediatric Febrile Neutropenia with Cancer

**DOI:** 10.1093/ofid/ofab466.266

**Published:** 2021-12-04

**Authors:** Muayad Alali, Allison Bartlett, Lara Danziger-Isakov, Lara Danziger-Isakov

**Affiliations:** 1 Indiana university, Carmel, Indiana; 2 University of Chicago, Chicago, IL; 3 Cincinnati Children’s Hospital Medical Center, Cincinnati, OH

## Abstract

**Background:**

Fever with neutropenia (FN) is common and the timing of antibiotic cessation in patients without an identified fever source is uncertain. Absolute neutrophile count (ANC) recovery has been used clinically to represent bone marrow recovery (BMR) but other options should be considered. We hypothesized that absolute monocyte count (AMC), and absolute phagocyte count (APC) are more sensitive, and an earlier safe marker of antibiotic cessation (AC) compared with ANC

**Methods:**

A retrospective review was performed for FN episodes (FNEs) at UCM Comer Children’s Hospital between 2009 and 2016 in pediatric oncology patients. Eligible FNEs who were a febrile for 24 hours, had no bacterial source identified at time of AC, and did not receive chemotherapy 10 days following AC. Ten-day post-AC outcomes, length of stay and cost were assessed and compared among different BMR parameters (ANC vs AMC).

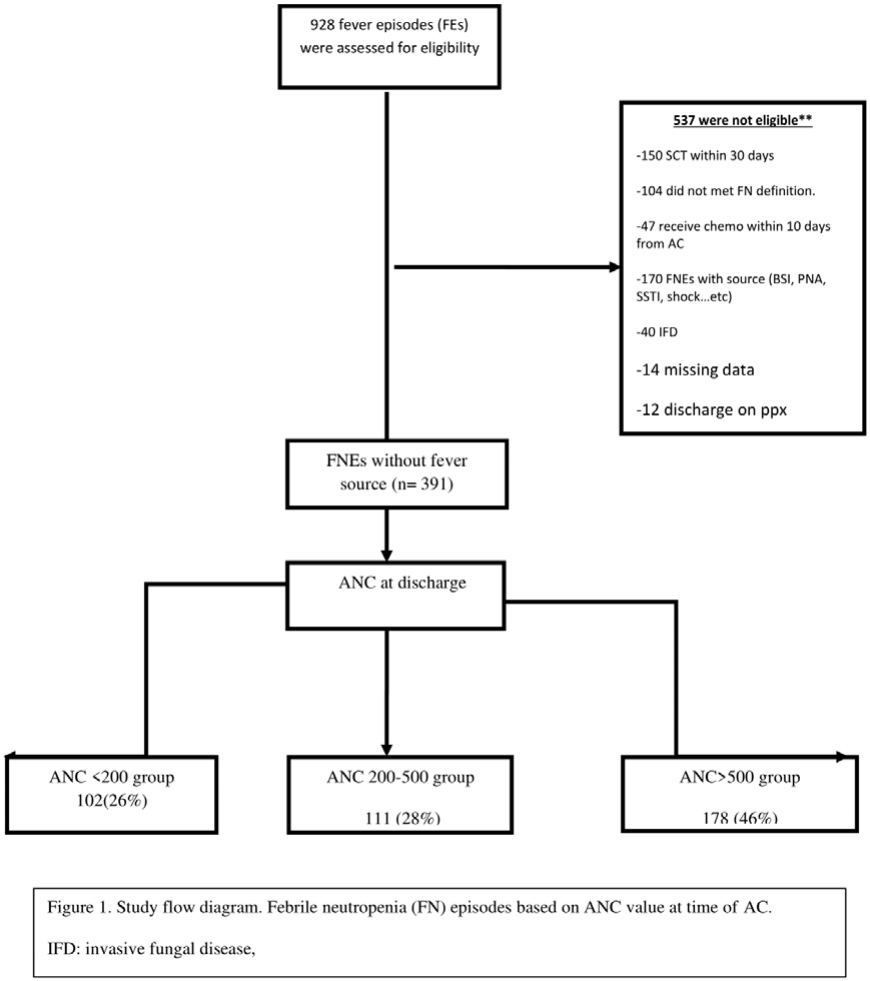

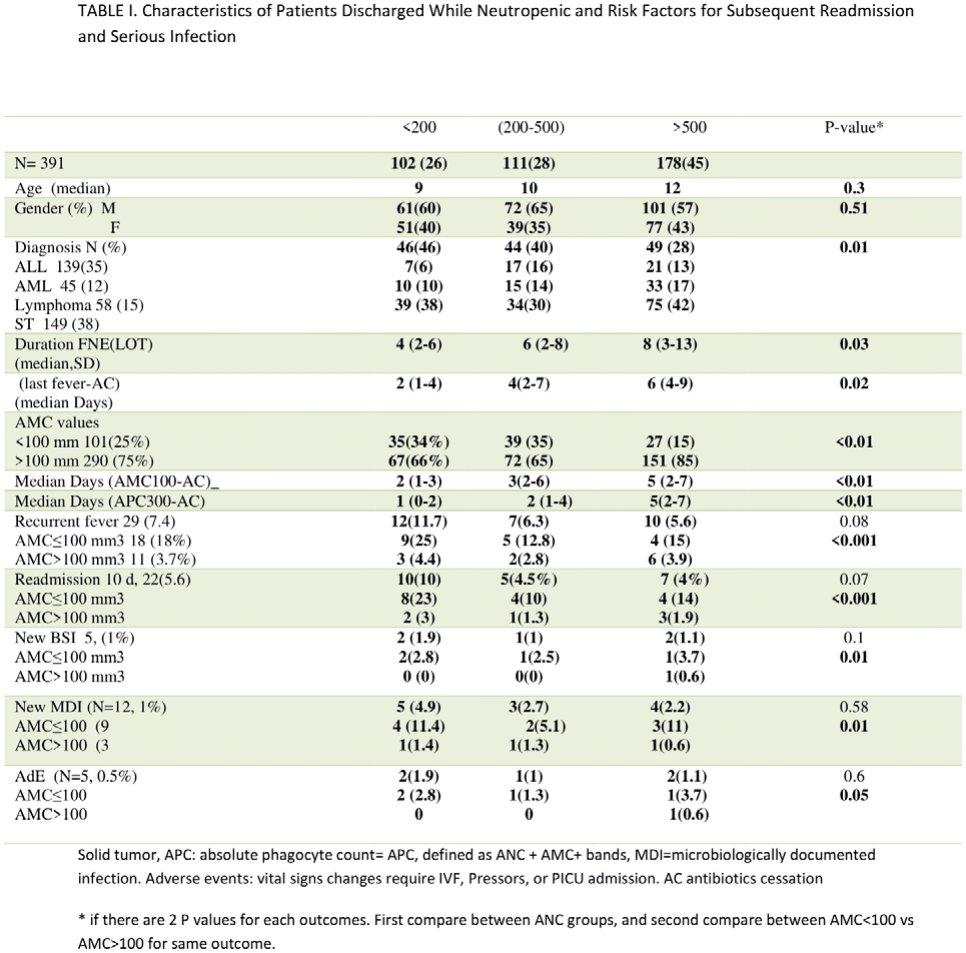

**Results:**

A total of 928 FN episodes (FNEs) were identified. 391 eligible FNEs occurred in 235 patients. Three groups were compared based on ANC (cells/μL) at the time of AC : < 200 in 102 (26%), 200-500 in 111 (28%), and >500 /uL in 178 (46%) (Figure1) with an overall ten-day recurrent fever rate 7.4% (29/391) and readmission rate of 5.6% (22/391). No significant differences in recurrent fever rates were identified among 3 ANC groups (11.7%, 6.3% and 5.6% respectively, P=0.08) and readmission (10%,4.5%, 4%, respectively; P=0.07)(Table 1).In subset analysis of AMC for each ANC group, patients with AMC >100 at AC have favorable outcomes, regardless ANC threshold (P< 0.01) (Table 1). Median of length of stay of FN was 3 days shorter using AMC >100/uL for BMR compared with any threshold of ANC (P< 0.01) and decrease overall FN cost stay (P< 0.01) (Table 2). Similar analysis show APC >300/uL at time of AC has favourable outcomes and decrease LOS regardless ANC threshold (data not shown here).

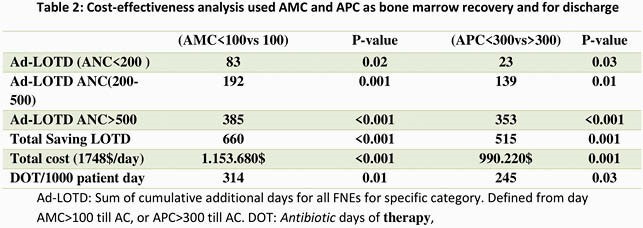

**Conclusion:**

Our results suggest that a AMC > 100 /uL regardless of ANC/uL, is a safe threshold value for empiric AC and discharge. This approach may shorten length of stay, reduce burden of cost of febrile neutropenia cost and potential long term antibiotics side effects.

**Disclosures:**

**Lara Danziger-Isakov, MD, MPH**, Ansun (Individual(s) Involved: Self): Scientific Research Study Investigator; Astellas (Individual(s) Involved: Self): Scientific Research Study Investigator; Merck (Individual(s) Involved: Self): Consultant, Scientific Research Study Investigator; Pfizer (Individual(s) Involved: Self): Scientific Research Study Investigator; Shire (Individual(s) Involved: Self): Consultant, Scientific Research Study Investigator; Viracor: Grant/Research Support

